# The State of Evidence in Patient Portals: Umbrella Review

**DOI:** 10.2196/23851

**Published:** 2020-11-11

**Authors:** Marcy G Antonio, Olga Petrovskaya, Francis Lau

**Affiliations:** 1 University of Victoria Victoria, BC Canada; 2 University of Alberta Edmonton, AB Canada

**Keywords:** CERQual, evidence-based practice, GRADE, patient portals, personal health records, systematic reviews, umbrella review

## Abstract

**Background:**

Patient portals have emerged as a recognized digital health strategy. To date, research on patient portals has grown rapidly. However, there has been limited evaluation of the growing body of evidence on portal availability, use, clinical or health behavior and outcomes, and portal adoption over time.

**Objective:**

This paper aims to comprehensively consolidate the current state of evidence on patient portals using the umbrella review methodology, introduce our approach for evaluating evidence for quantitative and qualitative findings presented in included systematic reviews, and present a knowledge translation tool that can be used to inform all stages of patient portal adoption.

**Methods:**

For this study, a modified version of the Joanna Briggs Institute umbrella review method was used. Multiple databases were searched for systematic reviews focused on patient portals, and the final sample included 14 reviews. We conducted a meta-level synthesis of findings from quantitative, qualitative, and mixed methods primary studies reported in systematic reviews. We organized the umbrella review findings according to the Clinical Adoption Meta-Model (CAMM). Vote-counting, GRADE (Grading of Recommendations, Assessment, Development, and Evaluations), and CERQual (Confidence in the Evidence from Review of Qualitative Research) were used to assess the umbrella review evidence.

**Results:**

Our knowledge translation tool summarizes the findings in the form of an evidence map. Organized by the CAMM categories, the map describes the following factors that influence portal adoption and effects over time: patient contexts, patient's interest and satisfaction, portal design, facilitators and barriers, providers' attitudes, service utilization, behavioral effects, clinical outcomes, and patient-reported outcomes. The map lists the theories and mechanisms recognized in the included portal research while identifying the need for business models and organizational theories that can inform all stages of portal adoption. Our GRADE and CERQual umbrella review evaluation resulted in the majority of evidence being rated as moderate to low, which reflects methodological issues in portal research, insufficient number of studies, or mixed results in specific focus areas. The 2 findings with a high rating of evidence were patients' interest in using portals for communication and the importance of a simple display of information in the portals. Over 40 portal features were identified in the umbrella review, with communication through secure messaging and appointment booking mentioned in all systematic reviews.

**Conclusions:**

Our umbrella review provides a meta-level synthesis to make sense of the evidence on patient portals from published systematic reviews. Unsystematic and variable reporting of portal features undermines the ability to evaluate and compare portal effects and overlooks the specific context of portal use. Research designs sensitive to the social, organizational, policy, and temporal dimensions are needed to better understand the underlying mechanisms and context that leverage the identified factors to improve portal use and effects.

## Introduction

### Background

Internationally, there has been an increasing effort to engage patients and consumers in their own health care using information and communication technology. The COVID-19 pandemic significantly stimulated the adoption and use of information and communication technology in primary care and outpatient clinics to facilitate remote visits, distant monitoring, and communication during the period of social distancing, particularly for patients living with chronic conditions [1-4]. These current events will further motivate various stakeholders to revisit the importance of eHealth tools, including electronic patient portals. A recent example from Canada is the province of Alberta that launched 2 patient portals in 2019, with a plan to gradually expand functionalities and patient engagement [5,6]. Since April 2020, patients and providers tested for COVID-19 across Alberta are able to access their test results online via MyHealth Records [5].

Countries such as Canada, the United Kingdom, and the United States have created national consumer digital health strategies and programs to encourage greater patient and consumer interactions with their health care providers through a variety of digital health solutions such as the patient portal [7-9]. For example, Infoway, an organization promoting Canada’s health strategy, has funded several portal implementation projects over the last decade and produced benefit evaluation reports [8].

Patient portal is a digital health tool managed by a health organization to provide patients with secure online access to their own health information such as laboratory results, doctor’s notes, and medication lists; care services such as appointment booking and reminders; and communication with their health care providers via secure messaging from anywhere via the internet [10]. It is also known as a tethered personal health record, which is a web-based interface linked to an electronic health record (EHR) where patients can view and interact with their health care data and providers [11].

### Comparison With Prior Work

Despite widely acknowledged portal benefits, the empirical evidence on patient portals reflects important challenges and context-dependent outcomes of portal use. For example, a recent review examining the behavioral and clinical outcomes associated with portal use reported improved patient understanding of their health conditions and medication adherence while noticing modest or no effects on diabetes and hypertension biophysiological indicators [12]. Our previous review demonstrated that portal technology may inadvertently create health equity concerns by not paying sufficient attention to the social determinants of health during portal implementation [13].

Research on patient portals, including primary studies (PSs), systematic reviews (SRs), and meta-level reviews, has been rapidly growing. Most SRs focus on *specific* health conditions [14], patient populations [15], aspects of portal use and its effects or impact [16-18], or on a *select* study design, for example, randomized controlled trials (RCTs) using a portal as an intervention [16]. To date, 2 meta-level reviews have been published that summarize the findings on patient portals reported in SR papers [19,20]. van Mens et al [19] used the Clinical Adoption Framework to “map relationships between *determinant and outcome category*” from 19 SRs retrieved up to early 2018. Among the limitations of their review, van Mens et al [19] named inadvertent inclusion of duplicate PSs and the impossibility of evaluating the strength of evidence. The other review of 20 SRs summarized the methods (ie, study design), types of effects, and benefits of digital health interventions up to 2016 [20].

In addition to focusing on portal technology, both meta-level reviews [19,20] and several SRs [21-23] reported combined findings on a variety of eHealth tools, which may limit the reader’s ability to discern portal-specific effects. Further, SRs may not always explicitly assess the quality of included PSs, thus potentially giving equal weight to findings characterized by various degrees of empirical support. When quality appraisals are included, the focus is often on RCTs and not broadened to other study designs [12,24]. Further, to the best of our knowledge, no published reviews summarizing evidence on patient portals have evaluated the strength of generated evidence as the concluding step of their review process. The limited quality assessments of the included studies and the absence of evaluations of the strength of outcomes or evidence offer readers little guidance on interpreting blanket statements of mixed and inconclusive results. To sum up, the various types of portal reviews that have been conducted to date present some substantive and methodological limitations mentioned earlier, thus providing an opportunity for our umbrella review.

### Goal of This Study

Our contributions are threefold. The first is substantive: this umbrella review comprehensively consolidates the current state of evidence about patient portals. The second is methodological: we included a wide range of high-quality SRs that were specifically focused on portal technology, eliminated duplicate PSs, and appraised the quality of umbrella review quantitative and qualitative evidence using modified GRADE (Grading of Recommendations, Assessment, Development, and Evaluations) and CERQual (Confidence in the Evidence from Review of Qualitative Research) criteria, thus presenting the relative strength of each umbrella review finding. The third contribution is knowledge translation, where our findings in the form of an evidence map can provide guidance for organizations in their patient portal adoption efforts. This is especially important in the Canadian context as many jurisdictions are actively pursuing patient portals at this time.

## Methods

### Objectives and Questions

The objectives of this umbrella review are to summarize the current state of evidence in patient portals based on published SRs and to create an evidence-based knowledge translation tool for the adoption of this technology. The questions addressed in this umbrella review are as follows:

1. What are the characteristics of the patient portals being introduced and used in different settings?
2. What are the system-related, health care provider–related, and patient-related factors that influence the introduction, use, and impact of patient portals?
3. What is the impact of patient portals on clinical outcomes of care?

### Methodology

Our methodology is detailed in a published protocol [25] registered and updated in PROSPERO (International Prospective Register of Systematic Reviews; PROSPERO registration number CRD42018096657). We employed the Joanna Briggs Institute (JBI) umbrella review method [26] with *modifications* [25]. Overall, our umbrella review attempted to adhere to best practice methodological recommendations outlined by Pollock et al [27] and Smith et al [28].

#### Search Strategy and Inclusion or Exclusion Criteria

In April 2018, the original search was conducted in 9 databases: MEDLINE, EMBASE, CINAHL Plus with Full Text, Web of Science Core Collection, Scopus, the Cochrane Database of Systematic Reviews, the PROSPERO registry, the JBI Database of Systematic Reviews and Implementation Reports, and Proquest Dissertations and Theses. An updated search in MEDLINE was conducted in November 2019 to identify the SRs published since the initial search. The complete search strategy is provided in Multimedia Appendix 1. The key inclusion criterion was specific to SRs focused on patient portals (irrespective of population groups and study designs) and published since 1990 in English. The SRs that were excluded were those with multiple eHealth technologies, standalone (ie, not tethered) personal health records, those focused on low- and medium-resource countries and thus contextually unique, reviews of reviews, scoping and integrative literature reviews, and reviews that do not provide a complete list of included PSs or designs.

#### Review Selection and Critical Appraisal

Citations were imported to Covidence. Two researchers independently screened titles and abstracts and then full-text articles against the inclusion and exclusion criteria. Discrepancies were resolved by consensus between 2 researchers and/or by a third researcher. The methodological quality of each SR was independently assessed by at least two researchers using the JBI critical appraisal checklist for SRs consisting of 11 questions [26]. Upon reaching a consensus among all researchers, low-quality SRs with a cutoff point <6 were eliminated.

**Figure 1:**
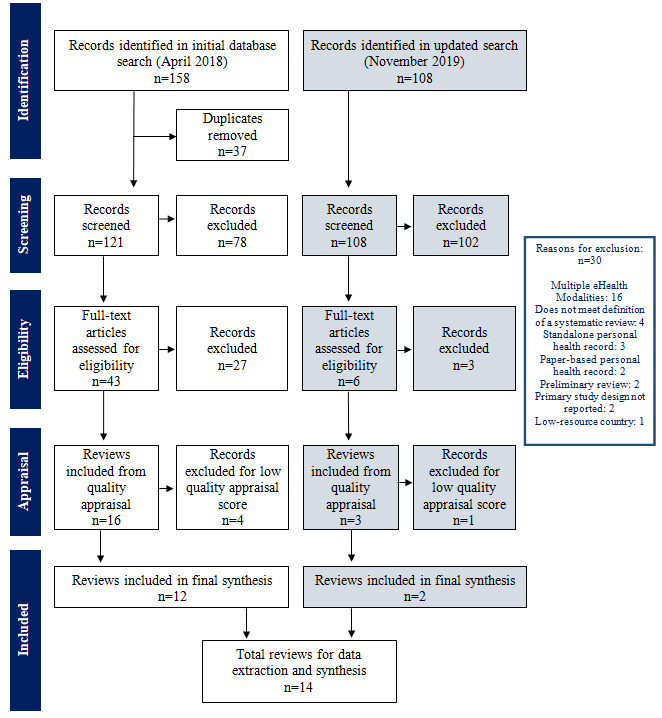
The PRISMA (Preferred Reporting Items for Systematic Reviews and Meta-Analyses) flow chart.

Our initial database search for SRs yielded 158 citations. After eliminating duplicates and screening for relevant titles and abstracts, we retained 40 SRs for full-text assessment. By applying the inclusion or exclusion criteria, we identified 16 SRs for critical appraisal. An updated search in November 2019 identified 108 citations, yielding 6 SRs for full-text assessment, 3 of which underwent critical appraisal (the list of excluded reviews from the full-text assessment is provided in Multimedia Appendix 2 [14-18,24,26,29-36]). The 19 SRs included in critical appraisal had scores that ranged from 3 to 11, with 6 reviews scoring 10 or 11 (Multimedia Appendix 2). Five SRs with scores <6 were excluded, leaving 14 reviews as the final selection for synthesis [14-18,24,29-36]. Figure 1 shows the Preferred Reporting Items for SRs and Meta-Analyses flow diagram for the selection process.

#### Analysis and Synthesis

In this paper, we refer to *the findings* at 3 different levels. PS findings are the individual findings reported by the SR authors in the results section and appendices of the SRs. In turn, SR findings represent the synthesis by SR authors reflecting how they combined PSs. The umbrella review findings are our synthesis of the PS findings reported in included SRs and of any conceptualizations or models advanced as the outcome in the included SRs.

**Figure 2:**
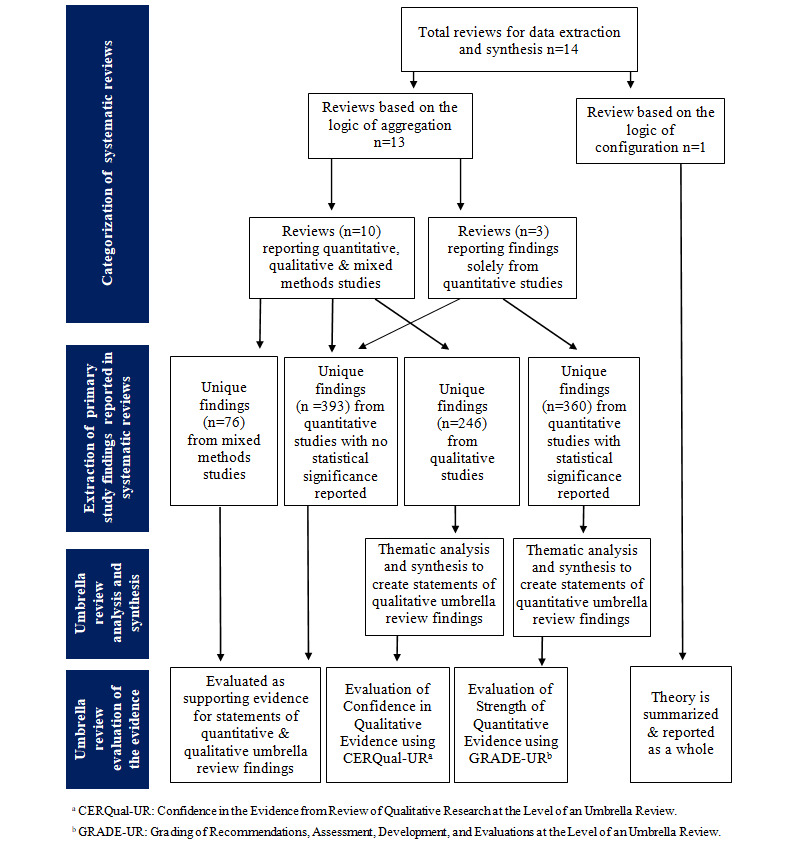
Data Extraction flow chart.

Initially, we categorized the included SRs according to the logic underpinning their approach to synthesis [25,37]: reviews following the aggregation logic (n=13) in which the SR authors reported outcomes based on the summary of individual PS findings and reviews following the configuration logic (n=1) where the SR authors' analysis combined findings from PSs to articulate a theory. Figure 2 shows our categorization of the included SRs for data extraction and synthesis. We grouped all included *aggregation* SRs into (1) group A: purely quantitative reviews (n=3) where the SR authors only reported on findings from quantitative PSs, and (2) group B: reviews with a mix of quantitative, qualitative, or mixed methods PSs (n=10). There were no purely qualitative reviews (ie, those with exclusively qualitative PS design).

One researcher extracted all the data, and 100% of the outputs were validated by at least one other researcher, with discrepancies resolved by discussions among the 3 researchers. All relevant PSs, their design, sample size, and all findings, as reported by the SR authors were extracted into Excel tables. Duplicate findings from duplicate PSs were removed to manage an overlap among the reviews. Data were synthesized into themes and then a smaller number of domains and statements. No statistical meta-analyses or subgroup analyses were performed due to heterogeneity of the study design.

As a precursor for rating the strength of umbrella review quantitative evidence and the confidence in umbrella review qualitative evidence, our 2 main data extraction documents were quantitative and qualitative Excel tables, respectively. The headings and/or introductory sentences in the paragraphs describing findings or results in each SR were used for initial domain categorization. Domains were further developed through an iterative process of cross-checking with the Clinical Adoption Meta-Model (CAMM) framework, sorting within Excel documents, and weekly analysis meetings among the researchers.

Our quantitative data extraction table included those PSs from SRs (both group A and group B), where the SR authors referenced statistical significance (*P* values, significance, and confidence intervals) of PS findings. Our qualitative data extraction table included those PSs from SRs in group B, where the SR authors provided sufficient information about the study design for the reader to classify those PSs as qualitative. After removing duplicates, the quantitative table included 360 unique individual findings from 10 SRs [14-17,30-35], and the qualitative table included 246 unique findings from 10 SRs [14,15,17,24,29-31,33,35,36]. Three SRs that were part of our umbrella review included papers that were a mix of PSs and reviews [17,24,35]. As an additional strategy to manage duplicates, we only extracted data from the PSs included in these SRs.

In addition, to extract all other data not fitting the 2 aforementioned tables, we created mixed methods and quantitative descriptive Excel data extraction documents, similar to the designs of PSs as reported in SRs. Our mixed methods table included 76 unique findings from mixed methods PSs from SRs in group B. The quantitative descriptive table included 393 unique findings from quantitative PSs from SRs in both groups A and B for which no statistical data or significance were provided by the SR authors, and which were unsuitable for evaluating the strength of evidence.

#### Rating the Umbrella Review Evidence

As the concluding step, 2 researchers independently assessed the strength of evidence for quantitative umbrella review finding statements and the confidence in the evidence for qualitative umbrella review finding statements. For this purpose, we developed meta-level umbrella review tools, GRADE-UR (Grading of Strength of Evidence for Quantitative Research at the Level of an Umbrella Review) and CERQual-UR (Grading of Confidence in the Evidence of Qualitative Research at the Level of an Umbrella Review), by applying a voting-counting method [38] and adapting GRADE [39-41] and CERQual [42-44] SR evaluation tools. The methodological approach was conceptualized independent from the GRADE working group. The GRADE-UR and CERQual-UR acronym was created by the authors of this paper to reflect an adaptation of GRADE and CERQual.

The GRADE-UR tool rates the strength of evidence as high, moderate, low, or insufficient [39-41]. Briefly, GRADE-UR evaluation of the quantitative finding statements is based on the following information: the SR authors' critical appraisal of PSs, the PS sample sizes, the number of RCTs, reporting of statistical significance, the number of PS findings that agree with the umbrella review finding statement, and the outcome measures used in PSs. The CERQual-UR tool rates confidence in the evidence as high, moderate, low, or very low. CERQual-UR evaluation of the qualitative finding statements is based on the following information: specific questions from our critical appraisal using the JBI checklist, SR methodological features, and their presentation of the results. A further description of GRADE-UR and CERQual-UR can be found in Multimedia Appendix 3 [26,38-44].

To the best of our knowledge, no tools are currently available for rating the evidence in reviews that synthesize mixed methods and findings from quantitative studies reported in SRs without any reference to statistical analysis; thus, we were unable to assess umbrella review evidence synthesized from our respective Excel tables. However, we compared umbrella review findings from mixed methods or quantitative descriptive PSs with the umbrella review findings from quantitative and qualitative PSs. We looked for differences and correspondences with the goal of formulating any additional umbrella review finding statements arising from mixed methods or quantitative descriptive findings and not subject to GRADE-UR or CERQual-UR evaluation. This information can be found in Multimedia Appendix 4 [14-18,24,29-36].

#### Output of the Umbrella Review

The output of our umbrella review consists of 2 summary of findings and evidence profile tables, a narrative synthesis, and a knowledge translation tool in the form of an evidence map on patient portal use and impact. CAMM [45] underpins the categorization of these outputs. CAMM is a maturity model used to understand, describe, and explain the adoption of digital health technologies through the preadoption, early adoption, and mature adoption stages [45]. It is a temporal *adoption* model with 5 dimensions: availability, system use, clinical/health behavior, outcomes*,* and time. In this review, adoption refers to the planning, implementation, utilization, and support of a patient portal. *Availability* refers to making the patient portal accessible to users. *Use* refers to patterns of user interaction and experience with the portal. *Clinical/health behavior* refers to changes in user behaviors from interacting with the portal. *Outcomes* refers to the health impact from portal use. *Time* refers to the transition periods across the 4 dimensions [45].

## Results

### Review Characteristics

The population, objectives, design, and context of the 14 reviews are described below and further summarized in Multimedia Appendix 2. All 14 reviews were published between November 2012 and November 2019, 11 of which were published in 2015 or later. With duplicates removed, 280 unique PSs were identified across the 13 *aggregation* reviews, resulting in 1075 unique findings. We handled the findings of the only *configuration* review [18] holistically, without disintegrating into separate components. This realist review [18] proposed mechanisms for achieving portal outcomes.

#### Population

A total of 6 reviews focused on specific populations: people with diabetes [14,35] or other chronic conditions [17] and pediatric [15], vulnerable [32], and hospitalized [33] patients. Study participants included patients, family members, parents or guardians, and health care providers. A total of 7 reviews reported sample sizes of their included PSs, with sample sizes ranging from 5 participants in a qualitative study [33] to 529,605 in a cross-sectional study [31].

#### Objectives

Included SRs examined patient engagement [29], facilitators and barriers of portals use [36], meaningful use [17], health literacy [30], mechanisms for achieving portal outcomes [18], effects [17,24,31], and the impact of portals [16,35]. The findings from quantitative PSs reported in SRs pertained to portal enrollment or use levels by sociodemographic factors, the role of portal training, patient satisfaction and empowerment, clinical outcomes including screening rates and treatment adherence, and portal impact on health service utilization [14-17,29,31,32,34]. The findings from qualitative PSs reported in SRs tended to focus on barriers and facilitators; portal design; communication between providers and patients; perceived care quality; providers' concerns; and patient safety, empowerment, engagement, and satisfaction [14,15,17,24,29-31,35,36].

#### Study Design

Across the 13 *aggregation* reviews, the number of PSs ranged from 5 [16] to 143 [24], yielding 280 unique PSs of varied designs. The quantitative PSs were classified as 32 RCTs, 18 cohort studies, 8 time series and 16 cross-sectional studies, 3 surveys, 8 pre-post, 2 post only, 1 retro-audit, and 1 quasi-experimental (further information on the design of the PSs for each of the UR findings is provided in Multimedia Appendix 4). In these quantitative studies, data were generated through structured self-report questionnaires, system logs, administrative data sets, and patient medical records. None of the reviews included meta-analyses due to the presence of heterogeneous population groups and diverse measures reported. The designs or methods of data collection used in qualitative and mixed methods PSs encompassed interviews, questionnaires, focus groups, usability studies including observations, and case reports. Critical appraisal tools were used in 7 reviews [14-16,24,31,32,34,35]. Heterogeneity was explicitly discussed as an issue in 4 reviews [16,24,32,34]. Limitations recognized by SR authors included the variability in portal utilization measures [14], portal types and population [31], and portal definitions [33], low quality of reporting [35], limited variety of study designs [31,33], and the potential of underpowered studies [32].

Across the 13 *aggregation* reviews, we found limited application of models, theories, and frameworks as the conceptual foundation for evaluating patient portal use and outcomes. None of the included SRs applied models, theories, or frameworks as a guide for their review. Some authors briefly referenced models, theories, and frameworks as justification for the review or in their discussion, whereas others summarized the use of models and theories in the included PSs [29,31,36]. Goldzweig et al [31] found that 5 out of 21 PSs used a theory or model, and Irizarry et al [29] found that 11 of 120 PSs used a theoretical framework, but the SR authors did not elaborate on how these theoretical underpinnings related to the outcomes. One SR observed that the chronic care model was cited most often within PSs [29], and 2 SRs commented on how the model’s concept of self-management influences outcomes [14,24]. The following theories were referenced across the SRs: Roger's Diffusion of Innovation [29], activation theory [36], theory of coping and self-determination [36], and grounded theory [sic] [36]. Review authors recommended reconsidering the business model of patient care [34] and developing a framework to identify appropriate outcome measures for long-term portal use [36].

#### Context

A total of 10 reviews named the countries where the PSs were conducted; most were in the United States, with Europe, Australia, and Asia referenced [14-16,18,24,30,31,34-36]. A total of 8 reviews reported study settings, which varied from hospitals, clinics, group practices, and primary care, or a combination of settings [15,16,24,29,31,33,34,36].

### Characteristics of Patient Portals

Portal features were reported inconsistently and unsystematically across reviews (a summary of the portal features described in the reviews is provided in Multimedia Appendix 2). Two SRs itemized and compared specific patient portal features reported across each PS [31,33]. Other SRs summarized portal features in their results section or appendices as an overview of the intervention, a collective summary sentence, or in reference to individual findings. Some reviews did not report specific portal features in their findings, but described portal functions in general, in the introductory section.

In total, we identified 41 portal features, with a range across reviews from 3 [30] to 25 [31]. Secure messaging or communication and appointment booking were the 2 features mentioned in all reviews. Other common features that we identified were access to laboratory and test results, visit summaries, and medication renewals. In contrast, portal features that allowed patients to generate data through care plans, patient self-assessment tools, journals, and the ability to edit data were mentioned less frequently. Frequently used features in a pediatric portal included immunization records, secure messaging, and appointment scheduling [15]. The SR by Kelly et al [33] on inpatient portals highlighted patients’ desire to be able to view their daily schedule and information on medication dose, frequency, timing, administration, route, and side effects.

Seven reviews mentioned features that patients viewed as desirable but not commonly offered. These included proxy access, medication glossaries with photos, medication side effects and instructions, care goals or plans with feedback, symptom tracking, videoconferencing, portal access through onsite kiosks, voice recognition for older adults, and text messaging for quality assurance service [15,18,24,29-31,33]. Specific features desired by patients in an inpatient setting included hospital room number; health care provider names or photos; medical information on condition and what will happen next; recovery goals; and access to physician notes, operative reports, and test results [33].

### Summary of Umbrella Review Findings, Evidence Profile, and the Knowledge Translation Tool

Tables 1 and 2 display the umbrella review finding statements and the evidence profile. To understand how the strength of the evidence and the confidence in the evidence were evaluated, refer to Multimedia Appendix 3. We found few examples of high confidence in the evidence and no examples of high strength of evidence. High confidence means that additional studies are unlikely to generate new findings on account of the topic being relatively well researched, SRs being of high quality, and the findings representing the phenomenon accurately. Moderate strength of evidence indicates that the finding is likely but there are some deficiencies in the current evidence [41]. Moderate confidence in the evidence indicates that the findings reasonably represent the phenomenon [43]. Low to very low confidence indicates that the topic is under-researched, studies have methodological weaknesses or inconsistent findings, and new studies are likely to generate useful findings that can contradict existing evidence. Our umbrella review findings should be interpreted in this context, with the amount and quality of existing research represented in the included SRs and the consistency of findings playing an important role in the rating of evidence.

**Figure 3:**
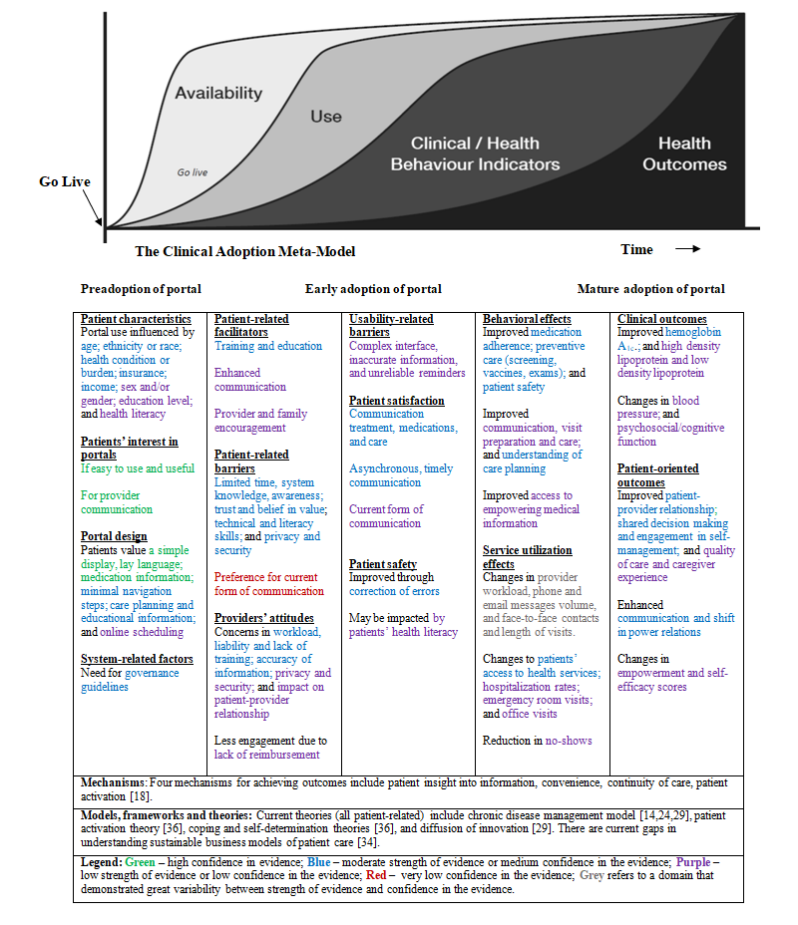
Evidence Map Across Portal Adoption Stages.

Our evidence map for patient portal adoption is presented in Figure 3. The map is a visual knowledge translation tool of the current state of evidence of patient portal use and impact. In this figure, the image of the 4 CAMM stages is from Price et al [45] and the 5 columns provided have been organized by the domain, and outcome or findings listed in Tables 1 and 2. In the remaining sections of our results, we present the umbrella review findings through the 4 stages of the map, while drawing attention to select strength of evidence and confidence in the evidence ratings.

**Table 1:** Summary of quantitative umbrella review findings and Grading of Strength of Evidence for Quantitative Research at the Level of an Umbrella Review evaluation of quantitative evidence.

Umbrella review domain and summary of quantitative findings statement (SR^a^ source)		Strength of the evidence according to the GRADE-UR^b^ criteria^c^
**Patient characteristics**		
	Patients with better controlled diabetes are more likely to enroll or use a portal as compared to other patients with diabetes [14,31,35].	Moderate
	Patients with private insurance in the US context are more likely to enroll or use a portal [14,15,31,34,35].	Moderate
	Patients with higher illness(es) burden or need are more likely to enroll or use a portal [14,15,31,34].	Moderate
	White people are more likely to enroll or use a portal [14,15,31,32,34].	Moderate
	Middle-aged people (≤65 years) are more likely to enroll or use a portal [14,17,31,32,34].	Moderate
	People who have a higher income are more likely to enroll or use a portal [14,15,31,35].	Moderate
	Males with diabetes are more likely to enroll or use portal as compared with females with diabetes [14,35].	Moderate
	Patients with higher health literacy are more likely to enroll or use a portal [14,17,30].	Low
	Females are more likely to access online information and use a portal [17,31,34].	Low
	People who have a higher education level are more likely to enroll in and use a patient portal [14,31,35].	Low
**Patient-related facilitators**		
	Patients are more likely to register and use a portal after portal-related education and training [32].	Moderate
**Patient satisfaction**		
	Patients who use patient portals report higher satisfaction with communication, treatment, medications, and care [16,31,34,35].	Moderate
**Behavioral effects**		
	Use of patient portals can increase adherence, mostly medication adherence across different patient populations [16,17,31,33,34].	Moderate
	Use of patient portals can improve screening, vaccinations, examinations, and/or care across different patient populations [31,34,35].	Moderate
	Use of patient portals can improve visit preparation and communication and information sharing between patients and providers [14,16,31,34].	Low
**Service utilization effects**		
	Health care provider’s workload related to contacts and messaging does not change with patient portal adoption [34].	Moderate
	Patients’ access to social support and mental health and testing services does not change with portal use [31,33].	Moderate
	Hospitalization rates do not change with patient portal use [16,31,34].	Low
	Emergency department visits do not change with patient portal use [16,31,34].	Low
	Phone or messaging volume received by health care providers does not change with patient portal use [16,17,31,34].	Low
	Patient portal use results in an increase in office, primary care, specialist, outpatient, or after-hour visits [15-17,31,34,35].	Low
	Patient portal use does not reduce no-show rates [17,34].	Low
**Clinical outcomes**		
	There is improvement in HbA_1c_^d^ levels for patients with diabetes who use patient portals [15-17,31].	Moderate
	There is improvement in LDL^e^, HDL^f^, cholesterol, or lipids for patients with diabetes who use patient portals [15,16,31,35].	Low
	There is no change in systolic and diastolic blood pressure for patients with diabetes or hypertension who use patient portals [16,31,35].	Low
	Psychosocial, cognitive function, BMI, symptom stability, and depression and anxiety status does not change across multiple patient populations who use patient portals [16,17,31].	Low
**Patient-oriented outcomes**		
	Patient empowerment and self-efficacy scores do not change with portal use [16,31].	Low

^a^SR: systematic review.

^b^GRADE-UR: Grading of Recommendations, Assessment, Development, and Evaluations at the Level of an Umbrella Review.

^c^Indicates the strength of the evidence and was calculated based on study limitations, directness, consistency, precision, and reporting of bias. The ratings are from high, moderate, and low. Any statements we evaluated as insufficient were moved to the supporting evidence tables in Multimedia Appendix 4.

^d^HbA_1c_: hemoglobin A_1c_.

^e^LDL: low-density lipoprotein.

^f^HDL: high-density lipoprotein.

**Table 2:** Summary of qualitative umbrella review findings and Grading of Confidence in the Evidence of Qualitative Research at the Level of an Umbrella Review evaluation of qualitative evidence.

Umbrella review domain and summary of qualitative findings statement (SR^a^ source)		Confidence in the evidence according to the CERQual-UR^b^ criteria^c^
**Patients’ interest in the potential of portals**		
	Patients are interested and satisfied in using patient portals if they are easy to use and useful [15,24,33].	High
	Patients are interested in using patient portals for communication and opportunity to message providers [17,30,33].	High
**Portal design and features**		
	Patients value information in patient portals that is easy to understand, written in lay or nonmedical language, transparent, and presented in a simple display [29,33].	High
	Patients want prescription refills, and hospitalized patients in particular want information on medication that includes dose, frequency, timing, administration, route, and side effects [29,33].	High
	Minimal navigation steps and educational information on specific laboratory results, medications, and allergies are important health equity and patient-friendly considerations [15,29,33].	Moderate
	The information within patient portals gives patients and parents a greater sense of control, involvement, understanding, and security in care planning [15,33,35].	Moderate
	Patients appreciate the scheduling function in patient portals, such as booking appointments online and scheduling, and daily planning in inpatient setting [15,29,33].	Low
**System-related factors**		
	Guideline development, framework for governance, and compliance with regulations are important for integrating patient portals into organizational processes [24,33].	Moderate
**Patient-related facilitators**		
	Use of patient portals is facilitated by the enhanced communication over traditional methods and positive patient-provider interactions and relationships [14,33,36].	Low
	Encouragement and instruction on patient portals offered by providers and families is a facilitator of portal use [14,29,36].	Low
**Patient-related barriers**		
	Patient barriers to portal use and enrollment include time, limited system knowledge, lack of awareness of patient portals and related features, and doubt or lack of belief in portal benefits or value [14,17,29,36].	Moderate
	Technical barriers to portal use and enrollment include type of interface, lack of technical or computer skills or training or support or literacy, lack of computer or internet access, and forgotten passwords [14,17,24,30,31,33,35,36].	Moderate
	Unauthorized access, privacy, security, and trust or confidentiality concerns are barriers to portal use and enrollment [14,15,24,29,30,33,36].	Moderate
	Patients’ lack of desire in enrolling and using portals relates to their preferences and satisfaction with existing means of communication [14,17].	Very low
**Providers’ attitudes and concerns**		
	Providers are concerned about liability and increases or changes in workload, and the lack of training, skills, and resources for using patient portals and prefer to have support staff screen messages [24,29,33].	Moderate
	Providers are concerned that the information contained in portals may overwhelm, cognitively overload, or increase patients’ anxiety and that patient-generated data may be inaccurate [24,29,33].	Moderate
	Providers perceive patient portals could encourage patient engagement, and secure messaging could support communication of complex information, while having concerns about impact on patient-provider relationships [24,29,33].	Low
	Providers are concerned about patient safety, privacy, and confidentiality and prefer control over access and authentication of users to protect the information in patient portals [24,33].	Low
	Lack of incentive and reimbursement may result in providers being less engaged with portals than patients may assume and instructing patients not to use [14,31].	Low
**Usability-related barriers**		
	Usability-related barriers which result in negative experiences and use of patient portals include: reminders and messages that are unreliable, have a slow response, or may not directly reach providers, and information that is inaccurate or difficult to locate due to complex navigation, visual layout, and language [14,29-31].	Low
**Patient satisfaction**		
	Online communication with providers outside their hours is preferred by patients and parents, as it is easier to understand, more convenient, supports accessing test results, and allows for timely and consistent responses [15,29,33,35].	Moderate
**Patient safety**		
	Patient portals enhance efficiency and patient safety when patients find and request correction of errors, especially medication errors [17,24,33,35].	Moderate
	Patients with limited health and computer literacy value portal use, but safe and effective use may be compromised by an inability to interpret results and having to take longer to complete patient portal tasks [29-31].	Low
**Behavioral effects**		
	Patient portals can facilitate access to medical information that can engage and empower patients to be confident in their self-management and current care [29,31,36].	Low
**Service utilization effects**		
	Patient portals can impact provider workload by increasing number of phone calls or emails or secure messaging and length of face-to-face visits [17,24,35].	Very low
**Patient-oriented outcomes**		
	Patient portals empower patients in shared decision making, prepare for visits, enable better expression of ideas and concerns, and encourage engagement in self-care and self-management [17,24,35].	Moderate
	Patient portals support communication, enhance discussions, and shift power relations between patients and providers [17,24,29,33,35].	Moderate
	Patient portals can improve quality of care and caregiver experience and reduce care burden [17,33].	Low

^a^SR: systematic review.

^b^CERQual-UR: Confidence in the Evidence from Review of Qualitative Research at the Level of an Umbrella Review.

^c^Indicates the confidence in the evidence and was calculated based on methodological limitations, coherence, relevance, and adequacy. Ratings are from high, moderate, low, and very low.

#### Who Adopts Patient Portals

Many SRs examined the characteristics of patients who use patient portals. Portal users are more likely to be middle aged (≤65 years) [14,17,31,32,34]; White (compared to Black, Hispanic and Asian) [14,15,31,32,34]; and have higher income [14,15,31,35], private insurance [14,15,31,34,35], higher education [14,31,35], or higher illnesses burden or service needs [14,15,31,34]. Females are more active adopters of patient portals when compared to males [17,31,34], except for those with lower socioeconomic status [34]. Among diabetes patients, males are more likely to enroll or use portals [14,35]. Patients with lower health literacy and numeracy skills are less likely to use portals [14,17,30].

#### Patients' Interest and Satisfaction in Portal Design

Patients are interested and satisfied in using patient portals if they are designed to be easy to use and useful [15,24,33]. The information in patient portals should be transparent and easy to understand, written in nonmedical language, and have a simple display [29,33]. To make portals more patient-friendly and attuned to variations in health literacy, there should be minimal navigation steps and educational information on specific laboratory results, medications, and allergies [15,29,33].

#### Factors to Consider in Increasing Portal Adoption by Patients

There are many barriers that impede the adoption of patient portals. Patients may forget their password, not have internet access, or lack the necessary interface or literacy and computer skills [14,17,24,30,31,33,35,36]. Portal use may also be discouraged when patients receive unreliable messages, encounter complex language, or have difficulty locating information within the portal [14,29-31]. Patients may also be unaware about portals, or have limited belief in portal benefits [14,17,29,36] and have privacy and security concerns [14,15,24,29,30,33,36]. Facilitation of portal adoption included encouragement from providers and family members [14,29,36] and training and education on portals [32].

#### System-Related Factors for Portal Implementation

A limited number of reviews addressed these factors. The lack of incentive or reimbursement may dissuade providers from promoting portal use to their patients [14,31]. Two SRs referenced the need for the development of guidelines and governance frameworks for integration of patient portals into organizational processes and to support compliance with regulations [24,33].

#### Health Care Providers’ Concerns on Portal Implementation

Multiple qualitative studies reported on providers’ attitudes toward portals [24,29,33]. The accuracy of patient-entered information and how portal information can increase patient anxiety or overwhelm, overload, and offend patients were repeated concerns of providers [24,29,33]. Providers preferred control over information to offset concerns about patient safety, privacy, and confidentiality [24,33]. Providers were also concerned about liability; change in workload; and the lack of training, skills, and resources for using patient portals [24,29,33].

#### Impact on Communication and Patient-Provider Relationships

Communication and influence on patient-provider relationships became a common thread throughout our evidence map. Some SRs found that patients were interested in the potential of using portals for communication with providers [17,30,33] and preferred the convenience, asynchronous aspect, and timeliness of communication afforded within patient portals [15,29,33,35]. Patients who used portals reported satisfaction with communication and the resulting treatment, medications, and care supported through portals [16,31,34,35]. However, some patients lack interest in portals, as they are satisfied with their current mode of communication [14,17].

Three SRs reported on providers’ interest in portals for encouraging patient engagement and communication of complex information with recognition of the potential impact on patient-provider relationships [24,29,33]. In addition, the evidence that portal use improved communication, information sharing, and patient-provider relationships [14,16,31,34] was rated as low.

#### Portal Use and Impact on Patient Outcomes and Behavioral Effects

Although patient portals may improve patient safety by having patients note and correct errors [17,24,33,35], safe and effective portal use may be compromised because of patients’ limited health and computer literacy [29-31]. Portal use may also reduce caregiver burden and improve the quality of preventive or follow-up care [17,33]. For clinical outcomes, evidence is limited and inconsistent. We found low strength of evidence for changes in blood pressure [16,31,35] and metabolic measures [15,16,31,35] with portal use. We found moderate strength of evidence for improvement in hemoglobin A_1c_ [15-17,31], preventive care [31,34,35], and medication adherence [16,17,31,33,34].

#### Impact on Service Utilization and Provider Workload

Limited number of studies have examined changes in the utilization of preventive or testing services [31,33], rate of hospitalizations [16,31,34] and emergency department visits [16,31,34], and no-show rates [17,34], all of which demonstrated low strength of evidence. Although a significant number of quantitative and qualitative PSs examined portal impact on provider workload, the evidence was inconsistent as to how patient portals may impact the number of contacts and face-to-face consults, phone volume, emails or messaging, and office visits [15-17,24,31,34,35]. We found very low confidence in the evidence that portals change provider workload and moderate strength of evidence for no change in workload. A similar pattern of variability was found within quantitative descriptive PSs not included in the aforementioned description: 10 found no change, 12 reported a decrease, and 20 reported an increase in provider workload.

#### Mechanisms

The review by Otte-Trojel et al [18] included in our analysis hypothesized 4 mechanisms for patient portals to achieve outcomes: patient insight, convenience, continuity of care, and patient activation [18]. Insights that patients gain from their online health information and EHR can improve communication, empowerment, understanding of one’s health condition, and adherence to treatment. Convenience is the time saved when patients have online access to providers and services. Care continuity improves patient-provider communication. Activation leads to empowerment through power balance and self-identity and to better self-care through improved relationships, trust, and availability of educational resources [18]. When we examined how similar concepts were referenced in the other included SRs, we found the following: low strength of evidence for association between portal use and changes to patient empowerment and self-efficacy scores [16,31]; moderate confidence in the evidence that portals could empower patients for self-care and shared decision making [17,24,35]; and moderate confidence in the evidence that portal information could provide patients and parents a greater sense of control, involvement, understanding, and security in care planning [15,33,35].

#### Time Dimension in Portal Use

The study by Grossman et al [32] was the only SR that reported in detail how portal use changed over time as a result of portal training and education. Bush et al [15] observed that longitudinal studies did not track portal usage over time. Other SRs did not seem to extract information on changes in portal use over time. Similarly, longitudinal changes in clinical outcomes were not highly represented in SRs, with 2 reviews referencing changes in outcomes over multiple time periods [31,35].

### Additional Findings From Mixed Methods or Quantitative Descriptive PSs

Data included in our mixed methods and quantitative descriptive extraction tables and not subject to the evidence rating provided additional support for the quantitative and qualitative umbrella review findings discussed earlier (Multimedia Appendix 4). In addition, noteworthy findings from this group of studies that are not found in our evidence tables include providers preferring emails focused on simple, self-limiting problems [24]; the importance of tailored messages sent to patients [29] and proxy access (family member access and caregiver access to information in the portal) [15,30,31,33]; and lower uptake of patient portals than initially anticipated [15,24]. Of note, proxy access and portal uptake are addressed only in a few PSs.

## Discussion

### Principal Findings

To the best of our knowledge, our umbrella review demonstrates the first attempt to adapt GRADE and CERQual processes and to develop GRADE-UR and CERQual-UR tools for evaluating evidence generated in an umbrella review. Moreover, our combined approach of evaluating the evidence and application of CAMM at the umbrella review level provides a novel approach for analyzing outcomes. In particular, we demonstrate how this approach can provide a more nuanced understanding of the evidence for common findings generated in portal research.

Our umbrella review provides an evidence map based on CAMM and the consolidated summary on the current state of evidence on patient portals and can be used to inform all stages of portal adoption. The map should be used in conjunction with the evidence tables to understand the strength of the available empirical support for different factors influencing patient portal adoption, use, and outcomes. In the next four paragraphs, we present key umbrella review findings and elaborate on how the map can be applied to address current knowledge gaps and across research, industry, policy, and practice.

The temporal aspect of CAMM suggests that portal adoption per se is not a guarantee of its effectiveness and that it should not be evaluated at a single point in time; rather, there are transitions from *interest* to *registration or enrollment*, *activation*, and then to *use or utilization* ending with *empowerment* in the best-case scenario. In our umbrella review, we found enrollment and use often being conflated, thus blurring the line between these 2 separate, yet very significant, dimensions of portal adoption.

We color coded different factors included in the evidence map to visually represent the strength of the current evidence. The colors can reveal not only existing knowledge gaps but also methodological assumptions made in some research on portals. For example, the evidence for the statement *portals change provider workload* was rated as very low, indicating that further research on this topic is likely to produce useful findings. However, the tables summarizing our findings also show that this topic has been extensively researched, but the studies exhibited great variability in the direction of the findings. This incongruence in the findings about changes to provider workload reveals a complex interplay of factors mobilized when technology is introduced into clinical practice and patients’ homes. Neither the types of studies that control for these factors (eg, RCTs) nor the study designs that simply explore participants’ satisfaction and perception are capable of shedding light on local organizational contexts that are often responsible for divergent portal outcomes.

The ideal use of the evidence map would be that each stakeholder looks across adoption stages and recognizes the interdependency among different factors, as the following examples suggest (italics indicate factors within the map). For example, for industry to support the promised long-term vision of improving clinical and patient-oriented outcomes, an *easy-to-use portal design* is required that does not introduce *usability barriers* at a later stage. In the policy realm, there is a need for *governance guidelines* that address both patients’ and providers’ *privacy and security concerns* to encourage trusting *patient-provider relationships*.

In addition, the evidence map reveals the areas of differing values that need to be considered within practice to achieve successful portal adoption. For example, patients are *interested in the communication* afforded by patient portals, whereas providers are concerned about the *increased workload* introduced by the new channels for communication. Further, portals are promoted as a tool for *patient empowerment*, whereas providers are concerned about *the loss of control* over information. With portals designed to serve more than one user group and to support their sometimes-divergent agendas, the success of portal technology hinges on acknowledging these different values and addressing these differences through the engagement of all relevant user groups.

### Limitations

There are a number of strengths to this umbrella review. First, the elimination of duplicate PSs from the included SRs provides a more accurate account of the reported findings. Second, the application of vote-counting, GRADE-UR, and CERQual-UR allows the direction, strength, and confidence of the evidence to be quantified and compared. Third, the evidence-based knowledge translation tool offers practical guidance to those involved in the planning, implementation, and support of patient portals. The evidence map seems promising as it helps to cast the use and impact of patient portals over time across preadoption, early adoption, and mature adoption stages while summarizing both key known success factors strongly supported by research and areas with low evidence base where more research is needed.

There are possible limitations to our search strategy. First, the literature on patient portals is varied in its coverage of eHealth modalities, inconsistent in defining portals, and evolving. Our search and selection strategy could have missed reviews that should be included. Second, we only focused on reviews published in English; those in other languages could have been missed. We also relied on the SR authors' identification of patient portals. However, in reviewing the SRs' reference list, we discovered that some SRs had included PSs that were broader in scope than the SR definition of patient portals. We accounted for these occasional discrepancies between the intended and actual SR focus during our GRADE-UR and CERQual-UR evaluation.

Our application of GRADE and CERQual at the meta-synthesis level could have been flawed, as there is little or no guidance available on how to appraise evidence synthesized across several SRs. In particular, we used SR authors’ reporting of statistical significance and *P* values to evaluate the strength of evidence. Many of the PSs included in the GRADE-UR evaluation had a limited sample size, and reporting on the power calculation of these studies was lacking; therefore, we were unable to evaluate these claims of statistical significance.

Similarly, we relied on SR authors’ reporting of the PS findings. When we suspected possible errors in SR authors’ reporting of findings, we consulted the original PS; in these cases, consensus among all umbrella review researchers was sought to determine how to address these individual discrepancies. However, this process was not exhaustive, and we did not cross-check all the findings between SRs and PSs.

### Comparison With Prior Work and Suggestions for Future Research

At the time of analyzing the included SRs, we noticed a certain tendency for not recognizing the findings from qualitative research as evidence worthy of being evaluated for its strength, which may reinforce the hierarchy of what is considered evidence. This observation might be explained by the lack of appropriate tools such as CERQual, which was developed recently and presents a counterpart to GRADE. The notion of *evidence* in the context of portal research should not be limited to a narrowly conceived evidence stemming from controlled studies. The role of RCTs is well recognized, and the strength of this design is acknowledged [20]; however, the findings and issues raised in our umbrella review and other reviews [46] call for a broader conceptualization of evidence. There are promising emergent qualitative designs that focus on the social, organizational, political, policy, and local context through emergent, ethnographic, and co-design approaches [47,48].

When examining portal-related outcomes, comparative studies to date have focused on biomedical measures for people with diabetes or hypertension and patient empowerment scores for general patient populations. We recommend further research with people living with other chronic conditions and comparative measures that communicate patients' values and perspectives [49]. This will involve greater integration of patient-oriented measures that can evaluate the outcomes that are of greatest interest to patients and experiences with their portal-enabled health care encounters.

Unsystematic reporting of portal features in the included SRs may reveal a mistaken assumption in some patient portal research that portal features are inconsequential for the outcomes. In comparative research, this can lead to a faulty comparison of portals that may be quite different from each other. We recommend that researchers report actual portal functionalities and technology characteristics. Ammenwerth et al [50] offer a simple yet useful portal taxonomy that includes 7 functionalities (access, remind, request, communicate, share, manage, and educate), which can be easily used when describing portal features to support comparisons across settings and studies.

Consistent with the findings of other SRs not included in our umbrella review, we found that the most extensive areas of research and evidence were on patient-related factors, namely, common barriers and facilitators [22,51], clinical and behavioral outcomes [20], and the role of patient demographic factors in portal adoption [19,20,22,46,51]. Health care provider–related factors were primarily focused on provider concerns [22,46,51], with a lack of examples on how these concerns can be addressed. SR reporting of a lack of guidelines and business models to guide portal implementation was the only statement we identified under system-related factors. Our umbrella review extends the understanding of these patient-, provider-, and system-related factors by reporting and rating the evidence at a meta-level. In addition, we identified current evidence gaps related to proxy access, portal uptake, and most significant to our review, the need for theoretical frameworks sensitive to system-level factors. We aimed to address this theoretical gap in applying CAMM to the development of our evidence map for patient portal adoption.

### Conclusions

Our umbrella review offers an organized knowledge translation tool on what is known about patient portals, the quality of the available evidence, and areas that require further work. The evidence map can be used to inform planning, implementing, and supporting the adoption of patient portals across research, industry, policy, and practice. Through our GRADE-UR and CERQual-UR approach, we demonstrated not only how to consolidate findings from SRs, including PSs of various designs, but also how to evaluate the strength and confidence in the evidence of findings from quantitative and qualitative studies. For many of the umbrella review findings, the quality of the evidence was rated as low. This suggests at least two interrelated conclusions. For many identified factors playing a role in portal success, their interactions and underlying mechanisms, especially over time, are still mostly unknown and invite new research. Along with this, we need a broader conceptualization as to what constitutes evidence. This calls for study designs and theoretical perspectives attentive to the contextual complexity of portal adoption.

## Appendix

Multimedia Appendix 1 Search strategy.

Multimedia Appendix 2 Exhibits for extraction of patient portal umbrella review.

Multimedia Appendix 3 Application of GRADE-UR (Grading of Confidence in the Evidence of Qualitative Research at the Level of an Umbrella Review) and CERQual-UR (Grading of Confidence in the Evidence of Qualitative Research at the Level of an Umbrella Review): An example.

Multimedia Appendix 4 Additional support for GRADE-UR (Grading of Confidence in the Evidence of Qualitative Research at the Level of an Umbrella Review) and CERQual-UR (Grading of Confidence in the Evidence of Qualitative Research at the Level of an Umbrella Review) findings.

## Abbreviations

CAMM: Clinical Adoption Meta-Model
CERQual: Confidence in the Evidence from Review of Qualitative Research
CERQual-UR: Grading of Confidence in the Evidence of Qualitative Research at the Level of an Umbrella Review
EHR: electronic health record
GRADE: Grading of Recommendations, Assessment, Development, and Evaluations
GRADE-UR: Grading of Strength of Evidence for Quantitative Research at the Level of an Umbrella Review
JBI: Joanna Briggs Institute
PS: primary study
RCT: randomized controlled trial
SR: systematic review
