# Correction: The State of Evidence in Patient Portals: Umbrella Review

**DOI:** 10.2196/32421

**Published:** 2021-08-16

**Authors:** Marcy G Antonio, Olga Petrovskaya, Francis Lau

**Affiliations:** 1 University of Victoria Victoria, BC Canada; 2 University of Alberta Edmonton, AB Canada

In “The State of Evidence in Patient Portals: Umbrella Review” (J Med Internet Res 2020;22(11):e23851) the authors added a clarification.

In the section “Rating the Umbrella Review Evidence,” two sentences have been added to the text to clarify that this work was not done under the guidance of the GRADE working group but was conceptualized independently of the group. In the originally published paper, the first paragraph of this section appeared as follows:

As the concluding step, 2 researchers independently assessed the strength of evidence for quantitative umbrella review finding statements and the confidence in the evidence for qualitative umbrella review finding statements. For this purpose, we developed meta-level umbrella review tools, GRADE-UR (Grading of Strength of Evidence for Quantitative Research at the Level of an Umbrella Review) and CERQual-UR (Grading of Confidence in the Evidence of Qualitative Research at the Level of an Umbrella Review), by applying a voting-counting method [38] and adapting GRADE [39-41] and CERQual [42-44] SR evaluation tools.

This has been corrected to:

As the concluding step, 2 researchers independently assessed the strength of evidence for quantitative umbrella review finding statements and the confidence in the evidence for qualitative umbrella review finding statements. For this purpose, we developed meta-level umbrella review tools, GRADE-UR (Grading of Strength of Evidence for Quantitative Research at the Level of an Umbrella Review) and CERQual-UR (Grading of Confidence in the Evidence of Qualitative Research at the Level of an Umbrella Review), by applying a voting-counting method [38] and adapting GRADE [39-41] and CERQual [42-44] SR evaluation tools. The methodological approach was conceptualized independent from the GRADE working group. The GRADE-UR and CERQual-UR acronym was created by the authors of this paper to reflect an adaptation of GRADE and CERQual.

The correction will appear in the online version of the paper on the JMIR Publications website on August 16, 2021, together with the publication of this correction notice. Because this was made after submission to PubMed, PubMed Central, and other full-text repositories, the corrected article has also been resubmitted to those repositories.

