# Supplementary material for: The State of Evidence in Patient Portals: Umbrella Review
Source: J Med Internet Res. 2020 Nov 11;22(11):e23851. doi: 10.2196/23851 (PMC7688386; doi:10.2196/23851)
Supplement: Multimedia Appendix 1 [file jmir_v22i11e23851_app1.pdf]

Multimedia Appendix 1
Search Strategy: April 2018

Ovid MEDLINE(R) Epub Ahead of Print, In-Process & Other Non-Indexed Citations, Ovid MEDLINE(R) Daily and Ovid MEDLINE(R) <1946 to Present>
--

1 patient portals/
2 ((patient* or consumer*) adj2 portal*).ti,ab,kf.
3 (portal hypertension or portal vein* or portal venous or ((proton or carbon) adj2 portal*)).mp.
4 2 not 3
5 1 or 4
6 (exp "Health Records, Personal"/ or "Patient Access to Records"/) and (electronic or online or internet or web or portal* or tethered).mp.
7 ((tethered adj3 record*) or eclinician or mychart).ti,ab,kf.
8 1 or 5 or 6 or 7
9 meta-analysis.pt.
10 (meta-anal$ or metaanal$).mp.
11 ((quantitativ$ adj3 review$1) or (quantitativ$ adj3 overview$)).mp.
12 ((systematic$ adj3 review$) or (systematic adj3 overview$)).mp.
13 ((methodologic adj3 review$1) or (methodologic adj3 overview$)).mp.
14 (integrat$ adj5 research).mp.
15 (quantitativ$ adj3 synthes$).mp.
16 ((qualitiative* adj3 (review* or overview)) or (meta-synthes* or metasynthes*)).mp.
17 or/9-16
18 review.pt. or (review$ or overview$).mp.
19 (medline or medlars or pubmed or index medicus or embase or cochrane).mp.
20 (scisearch or web of science or psycinfo or psychinfo or cinahl or cinhal).mp.
21 (excerpta medica or psychlit or psyclit or current contents or science citation index or sciences citation index or scopus).mp.
22 (hand search$ or manual search$).mp.
23 ((electronic adj3 database$) or (bibliographic adj3 database$) or periodical index$).mp.
24 (pooling or pooled or mantel haenszel).mp.
25 (peto or der simonian or dersimonian or fixed effect$).mp.
26 ((combine$ or combining) adj5 (data or trial or trials or studies or study or result or results)).mp.
27 or/19-26
28 18 and 27
29 17 or 28
30 (hta$ or health technology assessment$ or biomedical technology assessment$).mp.
31 technology assessment, biomedical/ or biomedical technology assessment/
32 30 or 31
33 29 or 32
34 8 and 33

Embase <1974 to Current>
----

1 (patient* adj2 portal*).ti,ab,kw.
2 (portal hypertension or portal vein* or portal venous or ((proton or carbon) adj2 portal*)).mp.
3 1 not 2
4 ((tethered adj3 record*) or eclinician or mychart).ti,ab,kw.
5 3 or 4
6 ((qualitiative* adj3 (review* or overview)) or (meta-synthes* or metasynthes*)).mp.
7 (meta-anal$ or metaanal$).mp.
8 ((quantitativ$ adj3 review$1) or (quantitativ$ adj3 overview$)).mp.
9 ((systematic$ adj3 review$) or (systematic adj3 overview$)).mp.
10 ((methodologic adj3 review$1) or (methodologic adj3 overview$)).mp.
11 (integrat$ adj5 research).mp.
12 (quantitativ$ adj3 synthes$).mp.
13 or/6-12
14 review.pt. or (review$ or overview$).mp.
15 (medline or medlars or pubmed or index medicus or embase or cochrane).mp.
16 (scisearch or web of science or psycinfo or psychinfo or cinahl or cinhal).mp.
17 (excerpta medica or psychlit or psyclit or current contents or science citation index or sciences citation index or scopus).mp.
18 (hand search$ or manual search$).mp.
19 ((electronic adj3 database$) or (bibliographic adj3 database$) or periodical index$).mp.
20 (pooling or pooled or mantel haenszel).mp.
21 (peto or der simonian or dersimonian or fixed effect$).mp.
22 ((combine$ or combining) adj5 (data or trial or trials or studies or study or result or results)).mp.
23 or/15-22
24 14 and 23
25 13 or 24
26 (hta$ or health technology assessment$ or biomedical technology assessment$).mp.
27 technology assessment, biomedical/ or biomedical technology assessment/
28 26 or 27
29 25 or 28
30 5 and 29

Cochrane Library (Cochrane Reviews and Other Reviews)

#1 [mh "patient portals"] or ((patient* or consumer*) near/2 portal*):ti,ab,kw
#2 ("portal hypertension" or "portal vein*" or "portal venous" or ((proton or carbon) near/2 portal*)):ti,ab,kw
#3 #1 not #2
#4 [mh "Health Records, Personal"] or [mh "Patient Access to Records"] and (electronic or online or internet or web or portal* or tethered):ti,ab,kw or (tethered near/3 record*):ti,ab,kw or (eclinician or mychart):ti,ab,kw
#5 #3 OR #4

CINAHL

(patient* n2 portal* OR ((MH "Patient Access to Records") and (electronic or online or web or portal*))) NOT ("portal hypertension" or "portal vein*" or "portal venous" or (proton or carbon) w2 portal*) OR tethered w3 record* or eclinician or mychart
AND
TI review OR ("meta analys*" or "meta-synthes*" or metanalysi* or metasynthes*) OR ((quantitative or qualitative or methodological or systematic) w3 (review or overview)) OR
PT("Meta Analysis" OR "Meta Synthesis" OR Review OR "Systematic Review")

PROSPERO

patient portals

Joanna Briggs via Journals@Ovid

Your Journals@Ovid
----
1 "jbi*".jn.
2 ((patient* or consumer*) adj2 portal*).tw. Or ((tethered adj3 record*) or eclinician or mychart).tw.
3 1 and 2

Scopus/Advanced Search

TITLE-ABS-KEY ((patient W/2 portal*) OR (tethered w/3 record*) OR eclinician OR mychart) AND TITLE-ABS-KEY ((systematic OR quantitative OR qualitative OR methodological) PRE/3 (review* OR overview*) OR "meta-analys*" OR metanalys* OR "meta-synthes*" OR metasynthes*)

Web of Science Core Collection/Advanced Search

TS=((patient near/2 portal*) or(tethered near/3 record*) or eclinician OR mychart) AND TS=(((systematic OR quantitative OR qualitative OR methodological) near/3 (review* OR overview*)) OR "meta-analys*" OR metanalys* OR "meta-synthes*" OR metasynthes*) NOT TS=("portal hypertension" or "portal vein*" or "portal venous" or ((proton or carbon) near/2 portal*))

Proquest Dissertations & Theses Global

(patient near/2 portal*) or (tethered near/3 record*) or eclinician or mychart
AND
(systematic OR quantitative OR qualitative OR methodological) near/3 (review* OR overview*) OR "meta-analys*" OR metanalys* OR "meta-synthes*" OR metasynthes*
