# Supplementary material for: The State of Evidence in Patient Portals: Umbrella Review
Source: J Med Internet Res. 2020 Nov 11;22(11):e23851. doi: 10.2196/23851 (PMC7688386; doi:10.2196/23851)
Supplement: Multimedia Appendix 3 [file jmir_v22i11e23851_app3.pdf]

Multimedia Appendix 3
Application of GRADE-UR and CERQual-UR: An example

We developed GRADE-UR by combining the Grading of Recommendations, Assessment, Development and Evaluation (GRADE) with vote counting method. The version of GRADE we used is based on the updated Guidance from the US Evidence-based Practice Centre (EPC) [39], and has been applied by Gibbons et al [40] to determine the strength of quantitative evidence in their review of consumer eHealth tools. The vote-counting method tallies the percent of positive-neutral-negative primary study findings by outcome as was done by Lau et al. [38] in their overview of health information system impacts. Our GRADE-UR tool rates the strength of evidence as high, moderate, low and insufficient based on five criteria: limitations, directness, consistency, precision and reporting of bias [39-41].

We developed CERQual-UR tool based on the Confidence in Evidence from Reviews of Qualitative Research method (CERQual) described by the GRADE Working Group to assess the level of confidence in findings from qualitative synthesis [41-44]. CERQual-UR evaluates the confidence in evidence as high, moderate, low or very low, based on four criteria: methodological limitations, coherence, relevance and adequacy.

Below we use a sample of two narrative statements synthesized as the findings of our umbrella review and demonstrate application of GRADE-UR and CERQual-UR to evaluate the strength of quantitative evidence and confidence in the qualitative evidence for these statements, respectively. The first statement, *Portal use is associated with improved HemoglobinA1c (HgA1c) level for patients with diabetes*, was based on data from 14 unique primary studies (PSs) across four systematic reviews (SRs) in our quantitative data extraction Excel, and represents "clinical outcomes" domain. The second statement, *Unauthorized access, privacy, security and trust or confidentiality concerns are barriers to portal use and enrollment*, was based on data from 11 PSs across seven SRs in our qualitative data extraction Excel, and represents *'barriers and facilitators*' domain. In order to be included in GRADE-UR and CERQual-UR evaluation, statements have to be supported by at least three PSs in the Excel table.

GRADE-UR: Evaluating Strength of the Quantitative Evidence at the Level of an Umbrella Review

**Sample Umbrella Review Finding Statement:**<br>***Portal use is associated with improved HbA1c level for patients with diabetes.***			
**Criteria**	**Operational Definition**	**Scoring Approach**	**Umbrella Review Score for the Above Statement**
Study limitations (low, medium, or high)	Methodological quality of PSs supporting the statement.	Three steps:<br>1) Examine SR to determine whether quality appraisal for the PS had been reported. When multiple SRs report QA on the same PS, use the more conservative evaluation.<br>2) If reported, classify the rating as *low*, *medium* or *high* for purpose of umbrella review. If not reported, then rate as *high*.	Evaluated as medium.<br>Rationale: majority of PSs had quality appraisals completed, and were scored as medium by umbrella review authors.

Directness (direct or indirect)	Whether the outcome variable (included in the statement) was measured directly or indirectly in PS (e.g., the type of instrument used).	*Direct* when the variable is measured in the most direct way, eg, blood glucose using the glucometer or patient satisfaction using a self-report questionnaire. Frequently, *indirect* is assigned; not only based on the type of instrument, but also the outcome variable is a proxy for the actual variable of interest; or if the relationship between the independent variable (portal) and dependent variable (outcome) is likely to be mediated by other unknown variables.	<u>Evaluated as indirect.</u><br>Rationale: the relationship between portal use and HbA1c level are complex as multiple factors influence HbA1c.
Consistency (consistent or inconsistent)	Whether findings from different PSs examining the same outcome variable point in the same direction.<br>Preliminary steps include vote counting: a) assign positive, neutral or negative to each PS finding viz-a-viz the umbrella review finding statement and<br>b) determine the % of each	*Consistent* if 65% or more of PS findings point in same direction.<br>*Inconsistent* if less than 65% of PS findings are in the same direction.	<u>Evaluated as consistent.</u><br>Rationale: For our “positively” formulated umbrella review statement, PS findings vis-à-vis the statement are:<br>68.9% positive (point in the same direction as the statement)<br>31.3% neutral (no change)<br>0.0% negative (point in the opposite direction as the statement)
Precision (precise or imprecise)	The degree of certainty in a conclusion. Based on empirical support and the style of reporting of statistical information.	*Precise* if SRs included sufficient number of PSs (more than 3) *or* if at least 65% of PSs reported P-values or confidence interval.<br>*Imprecise* if majority of PSs only reported as 'statistical significance' without actual P-value.	<u>Evaluated as precise.</u><br>Rationale: 12 out of total 14 PSs had P-values or confidence intervals reported.
Reporting bias (undetected or suspected)	Whether SR assessed the risk of reporting bias.	*Undetected* if SR assessed bias and found none. If SR did not include information on reporting bias in included RCT PSs, assume *suspected*. For non-RCT PSs, evaluate assume not done, and rate as *suspected*.	<u>Evaluated as suspected.</u><br>Rationale: 6 RCT PSs (risk of bias: 2 low, 2 medium, 2 high); 8 non-RCTs.
**Calculate Overall Strength of the Evidence**			
**Definition** *(adapted from GRADE)*	**Scoring**		**Strength of Evidence**
Overall	Apply the GRADE summary rules to determine overall		<u>Evaluated as Moderate.</u>

strength of evidence based on the five criteria	GRADE-UR score for a given finding statement. Ratings range from high, moderate, low or very low.	Study limitations - Medium<br>Directness - Indirect<br>Consistency - Consistent<br>Precision - Precise<br>Reporting Bias – Suspected<br>*The reader would need to refer to a table of GRADE summary rules created by the umbrella review authors in order to understand how these criteria were transformed into the final score.*
**Conclusion**		
There is moderate strength of evidence that patient portal use is associated with improved HgA1c scores for patients with diabetes.		

CERQual-UR: Evaluating Confidence in the Qualitative Evidence at the Level of an Umbrella Review

**Sample Umbrella Review Finding Statement:** *Unauthorized access, privacy, security and trust or confidentiality concerns are barriers to portal use and enrollment.*				
**Criteria**	**Operational Definition** *(adapted from CERQual)[41-43]*	**Scoring:** Based on the JBI critical appraisal (JBI-CA) and other factors:<br>No concerns (1)<br>minor concerns (2)<br>moderate concerns (3)<br>serious concerns (4)	**Step one:** Assign score for **each** SR supporting the statement *(below is an example of scoring for one SR)*	**Step two:** Calculation of average for all PSs in all SRs [(#PSs x SR score) /#PSs]
Methodological Limitations	Extent to which there are methodological issues in the SR design.	Based on answers to JBI-CA questions 6 (*"Was critical appraisal conducted by two or more reviewers independently?"*), 7 (*Were there methods to minimize errors in data extraction?"*) and 9 (*"Was the likelihood of publication bias assessed?"*)[26]; and whether any formatting issues in SR were identified.	Scored as 4, serious concerns.<br>Q6->Unclear;<br>Q7->No;<br>Q9->Unclear	Scored as 3.2, moderate concerns.<br>Rationale: Of the 11 PSs in 7 SRs, 3 had no concerns (3x1) + 8 had serious concerns (8x4), divided by # of PSs = 3.2
Relevance	Extent to which the data/PS used in SR matches the SR proposed focus and research questions.	Based on answer to JBI-CA question 4 (*"Were the sources and resources used to search for studies adequate?"*)[26]; the match among SR research question and	Scored as 1, no concerns.<br>Q4->1 AND no other concerns	Scored as 1.5, minor concerns.<br>Rationale: Of the 11 PSs in 7 SRs, 5 had no concerns (5x1) + 4 had minor concerns

		search strategy, and the included PSs listed in SR reference list; and the match between included SRs and umbrella review focus.		(6x2), divided by # of PSs = 1.5.
Coherence	Extent to which SR findings are grounded in the data presented from PS in SR.	Based on answer to JBI-CA question 8 ("*Were the methods used to combine studies appropriate?")*[26]; and the match among SR objectives, research question and search strategy, and the synthesis of findings in the SR results section.	Scored as 1, no concerns. Q8 -> Yes AND no other concerns	Scored as 1.4, minor concerns. Of the 11 PSs in 7 SRs, 7 had no concerns (7x1) + 4 had minor concerns (4x2), divided by # of PSs = 1.4.
Adequacy	The scope and quantity of PS findings	Based on the quantity of PS findings and range of context or populations across PSs.	N/A	Evaluated as 1, no concerns. Rationale: Findings from 11 PSs with a mix of inpatient, general, pediatric settings and diabetic populations.
**Calculate Overall Confidence in the Evidence**				
**Definition** *(adapted from CERQual)*	**Scoring** *Ratings range from high (1), moderate (2), low (3) or very low (4)*		**Confidence score**	
Assessment of the extent to which the umbrella review statement is representative of the phenomenon of interest.	Calculate the average score based on the four criteria. Move down one level if the phenomenon in the statement is broad (ie, combines several related concepts from PS in SR).		Evaluated as 1.8, moderate concerns (3.2 + 1.5+ 1.4+1)/4= 1.8 Do not move down as it is a close fit between the umbrella review statement and PS findings.	
**Conclusion**				
Moderate confidence in the evidence that unauthorized access, privacy, security and trust or confidentiality concerns are barriers to portal use and enrollment.				
