# Supplementary material for: The State of Evidence in Patient Portals: Umbrella Review
Source: J Med Internet Res. 2020 Nov 11;22(11):e23851. doi: 10.2196/23851 (PMC7688386; doi:10.2196/23851)
Supplement: Multimedia Appendix 4 [file jmir_v22i11e23851_app4.pdf]

Multimedia Appendix 4

Table A: Support for Quantitative Umbrella Review Findings by Primary Study (PS) Findings from Systematic Reviews (SRs)

Umbrella review domain	Umbrella review quantitative finding statements [SR source]	Primary support used in GRADE-UR (Grading of Strength of Evidence for Quantitative Research at the Level of an Umbrella Review) process			Secondary support: Number of PS findings (A=Agreement; D=Disagreement with the UR finding statement)	
		Strength of umbrella review evidence[a]	PS design, number of PSs and sample size[b]	% of PS findings agreeing with umbrella review statement[c]	Mixed methods	Quantitative descriptive[d]
**Patient characteristics**						
	Patients with better controlled diabetes are more likely to enroll or use a portal as compared to other patients with diabetes [14,31,35].	Moderate	RCT (1, 104) non-RCT (8, 26217)	100.0% agreement 0.0% neutral 0.0% disagreement	none	1xA 1xD
	Patients with private insurance in the US context are more likely to enroll or use a portal [14,15,31,34-36].	Moderate	non-RCT (8, 935967)	100.0% agreement 0.0% neutral 0.0% disagreement	1xA	1xA
	Patients with higher illness(es) burden or need are more likely to enroll or use portal [14,15,17,31,34,36].	Moderate	RCT (1, 4500) non-RCT (7, 883797)	91.0% agreement 9.0% neutral 0.0%	1xA	5xA
	White people are more likely to enroll or use a portal [14,15,31,32,34,36].	Moderate	RCT (2, 4598) non-RCT (18, 464823)	88.0% agreement 12.0% neutral 0.0% disagreement	1xA	3xA 1xD
	Middle-aged people who are mid age (≤65 years) are more likely to enroll or use a portal [14,17,31,32,34,36].	Moderate	RCT (1, 4500) non-RCT (16, 570341)	75.0% agreement 15.0% neutral 10.0% disagreement	1xA	9A
	People who have a higher income are more likely to enroll or use a portal [14,15,24,31,35].	Moderate	non-RCT (13, 207014)	71.4% agreement 28.6% neutral 0.0% disagreement	none	5xA
	Males with diabetes are more likely to enroll or use portal as compared to females with diabetes [14,35].	Moderate	non-RCT (7, 21778)	71.4% agreement 28.6% neutral 0.0%	none	none

	Patients with higher health literacy are more likely to enroll or use portal [14,17,29,30].	Low	non-RCT (10, 83119)	85.7% agreement 14.3% neutral 0.0% disagreement	4xA 1XD (no difference)	1xA
	Females are more likely to access online information and use a portal [17,24,29,31,34-36].	Low	RCT (1, 4500) non-RCT (5, 929067)	83.3% agreement 0.0% neutral 16.7% disagreement	3xA	20xA 1xD (decreased) 2xD (mixed between genders)
	People who have a higher education level are more likely to enroll in and use a patient portal [14,15,31,35,36].	Low	RCT (1, 4500) non-RCT (9, 38924)	75.0% agreement 25.0% neutral 0.0% disagreement	none	2xA
**Patient-related facilitators**						
	Patient are more likely to register and use a portal after portal-related education and training [32].	Moderate	RCT (5,521) non-RCT (7, 71376)	58.30% agreement 41.7% neutral 0.00% disagreement	none	4xA
**Patient satisfaction**						
	Patients who use patient portals report higher satisfaction with communication, treatment, medications, and care [16,17,24,31,34,35].	Moderate	RCT (9, 3838) non-RCT (1, 529605)	64.3% agreement 35.7% neutral 0.0% disagreement	5xA	21xA
**Behavioral effects**						
	Use of patient portals can increase adherence, and mostly medication adherence, across different patient populations [16,17,24,31,33,34].	Moderate	RCT (5, 1181) non-RCT (4, 12990)	78.6% agreement 21.4% neutral 0.0% disagreement	none	9xA
	Use of patient portals can improve screening, vaccinations, examinations and/or care across different patient populations [24,29,31,34,35].	Moderate	RCT (3, 9039) non-RCT (2, 14782)	77.0% agreement 23.0% neutral 0.0% disagreement	1xA	3xA
	Use of patient portals can improve visit preparation and communication and information sharing between patients and providers [14,16,17,24,31,34-36].	Low	RCT (4, 4764) non-RCT (2, 541123)	88.9% agreement 11.1% neutral 0.0% disagreement	none	13xA

**Service utilization effects**						
	Health care provider's workload related to contacts and messaging does not change with patient portal adoption [24,29,31,34,35].	Moderate	RCT (3, 960)	85.7% agreement 14.3% disagreement (increase)	none	10xA 20xD (increase) 12xD (decrease)
	Patients' access to social support and mental health and testing services does not change with portal use [31,33].	Moderate	RCT (1, 129) non-RCT (2, 7417)	100.0% agreement 0.0% disagreement	none	none
	Hospitalization rates do not change with patient portal use [16,31,34].	Low	RCT (2, 164) non-RCT (1, 88642)	66.7% agreement 33.3% disagreement (reduced)	none	none
	Emergency department visits do not change with patient portal use [16,31,34,35].	Low	RCT (2, 859) non-RCT (1, 88642)	66.7% agreement 33.3% disagreement (increased)	none	1xD (increased)
	Phone or messaging volume received by health care providers does not change with patient portal use [16,17,24,31,34].	Low	RCT (5, 1890) non-RCT (3, 113530)	44.4% agreement 44.4% disagreement (increase) 11.2% disagreement (decrease)	none	8xA 2xD (increased)
	Patient portal use results in an increase in office, primary care, specialist, outpatient, or after-hour visits [15-17,24,31,34-36].	Low	RCT (7, 5888) non-RCT (6, 204719)	47.4% agreement 36.8% neutral 10.5% disagreement	1xA	4xA 1xD (decreased)
	Patient portal use does not reduce no-show rates [17,29,34].	Low	RCT (2, 271) non-RCT (1, 58942)	60.0% agreement 40.0% neutral 0.0% disagreement	none	2xA
**Clinical outcomes**						
	There is improvement in HbA1c[e] levels for patients with diabetes who use patient portals [15-17,31].	Moderate	RCT (6, 2995) non-RCT (8, 27662)	68.9% agreement 31.3% neutral 0.0%	1xA	none
	There is improvement in LDL[f], HDL[g], cholesterol, or lipids for patients with diabetes who use patient portals [15,16,31,35].	Low	RCT (4, 2407) non-RCT (3, 1290)	50.0% agreement 50.0% neutral 0.0% disagreement	none	none
	There is no change in systolic and diastolic blood pressure for patients with diabetes or hypertension who use	Low	RCT (7, 4139) non-RCT (2, 4210)	63.6% agreement 36.4% disagreement (improved)	none	none

	patient portals [16,31,35].					
	Psychosocial, cognitive function, BMI[h], symptom stability, and depression and anxiety status does not change across multiple patient populations who use patient portals [16,17,31].	Low	RCT (5, 1276) non-RCT (2, 369)	66.70% agreement 33.30% disagreement (improved)	none	none
**Patient-oriented outcomes**						
	Patient empowerment and self-efficacy scores do not change with portal use [16,31].	Low	RCT (6, 1017) non-RCT (1, 250)	89.0% agreement 11.0% disagreement (improved)	none	none

[a] Indicates the strength in the evidence, and was calculated using GRADE-UR (Grading of Recommendations, Assessment, Development, and Evaluations at the Level of an Umbrella Review) based on study limitations, directness, consistency, precision and reporting of bias. Ratings are from high, moderate and low.
[b] Design of the primary studies: whether they are a RCT or non-RCT. Number in brackets indicate the number of primary studies that have this design folLowed by the total number of participants across primary studies.
[c] Vote Counting: top number is % of findings that agree with statement, middle number indicates neutral finding to statement and bottom number indicates % of findings disagreeing with the statement. A neutral finding indicates one where the PS finding was reported as no change or no statistical significance was found.
[d] Quantitative descriptive findings originate from primary quantitative studies for which SR authors a) did not report statistical significance, or b) reported statistical significance, but did not have sufficient number of finding (i.e. greater than 2) to be included in GRADE-UR evaluation.
[e]HbA_{1c}: hemoglobin A_{1c}.
[f]LDL: low-density lipoprotein.
[g]HDL, high-density lipoprotein.
[h] BMI: body mass index

Table B: Support for qualitative umbrella review findings by primary study (PS) findings from systematic reviews (SRs)

Umbrella review domain	Umbrella review qualitative finding statements [SR source]	Confidence in the umbrella review evidence (based on CERQual-UR criteria)[a]	Number of PS findings (A=Agreement; D=Disagreement with the UR finding statement)		
			Qualitative	Mixed methods	Quantitative descriptive[b]
**Patients' interest in the potential of portals**					
	Patients are interested and satisfied in using patient portals if they are easy to use and useful [15,17,24,31,33,35].	High	13xA	1XA	7xA
	Patients are interested in patient portals for communication and opportunity to message providers [17,24,30,33].	High	11xA	1xA	3xA
**Portal design**					
	Patients value information in patient portals that is easy to understand, written in lay or non-medical language, transparent and presented in a simply display [29,33].	High	10xA	4xA	1xA
	Patients want prescription refills, and hospitalized patients in particular want information on medication that includes dose, frequency, timing, administration, route and side effects [15,17,29,31,33].	High	8xA	3xA	2xA
	Minimal navigation steps and educational information on specific laboratory results, medications and allergies are important health equity and patient-friendly considerations [15,29,33].	Moderate	7xA	43xA	1XA
	The information within patient portals gives patients and parents a greater sense of control, involvement, understanding and security in care planning [15,17,24,33,35].	Moderate	7xA	5xA	6xA
	Patients appreciate the scheduling function in patient portals, such as booking appointments online and scheduling, and daily planning in inpatient setting [15,29,33].	Low	4xA	none	none

**System-related factors**					
	Guideline development, framework for governance, and compliance with regulations are important for integrating patient portals into organizational processes [24,33].	Moderate	6xA	1xA	8xA
**Patient-related facilitators**					
	Use of patient portals is facilitated by the enhanced communication over traditional methods and positive patient-provider interactions and relationships [14,33,36].	Low	8xA	none	none
	Encouragement and instruction on patient portals offered by providers and families is a facilitator of portal use [14,29,36].	Low	5xA	none	8xA<br>1xD
**Patient-related barriers**					
	Patient barriers to portal use and enrollment include time, limited system knowledge, lack of awareness of patient portals and related features, and doubt or lack of belief in portal benefits or value [14,17,29,35,36].	Moderate	14xA	1xA	4xA
	Technical barriers to portal use and enrollment include type of interface, lack of technical or computer skills or training, support, or literacy, lack of computer/internet access, and forgotten passwords [14,15,17,24,29-31,33,35,36].	Moderate	12xA; 1xD	3xA	19xA
	Unauthorized access, privacy, security and trust/confidentiality concerns are barriers to portal use and enrollment [14,15,17,24,29-31,33,36].	Moderate	12xA	2xA,<br>1xD	5xA<br>1xD
	Patients' lack of desire in enrolling and using portals relates to their preferences and satisfaction with existing means of communication [14,17].	Very low	5xA	none	none
**Providers' attitudes and concerns**					
	Providers are concerned about liability and increases or changes in workload, and the lack of training, skills and resources for using patient portals, and prefer to have	Moderate	12xA	2xA	11xA

	support staff screen messages [24,29,33].				
	Providers are concerned that the information contained in portals may overwhelm, cognitively overload, or increase anxiety for patients, and that patient-generated data may be inaccurate [24,29,33,34].	Moderate	6xA	1xA 3xD	6XA
	Providers perceive patient portals could encourage patient engagement, and secure messaging could support communication of complex information, while having concerns about impact on patient-provider relationships [24,29,33].	Low	4xA, 2xD	none	none
	Providers are concerned about patient safety, privacy and confidentiality, and prefer control over access and authentication of users to protect the information in patient portals [24,33].	Low	5xA	none	109xA
	Lack of incentive and reimbursement may result in providers being less engaged with portals than patients may assume, and instructing patients not to use [14,31].	Low	4xA	none	none
**Usability-related barriers**					
	Usability-related barriers which result in negative experiences and use of patient portals include: reminders and messages that are unreliable, have a slow response, or may not directly reach providers, and information that is inaccurate or difficult to locate due to complex navigation, visual layout and language [14,15,29-31,35].	Low	13xA 1xD	7xA	3xA
**Patient satisfaction**					
	Online communication with providers outside their hours is preferred by patients and parents, as it is easier to understand, more convenient, supports accessing test results, and allows for timely and consistent responses [15,24,29,33,35].	Moderate	11xA	none	11xA

**Patient safety**					
	Patient portals enhance patient safety when patients find and request correction of errors, especially medication errors [17,24,33,35,36].	Moderate	6xA	none	8xA
	Patients with limited health and computer literacy value portal use, but safe and effective use may be compromised by an inability to interpret results and having to take longer to complete patient portal tasks [29,30].	Low	7xA	1xA	none
**Behavioral effects**					
	Patient portals can facilitate access to medical information that can engage and empower patients to be confident in their self-management and current care [17,29,31,34-36].	Low	10xA	1xA	5xA
**Service utilization effects**					
	Patient portals can impact provider workload, by increasing number of phone calls or emails/secure messaging and length of face-to-face visits [17,24,29,31,34,35].	Very low	5xA<br>4xD	none<br>(see Table A)	none<br>(see Table A)
**Patient-oriented outcomes**					
	Patient portals can empower patients in shared decision making, preparing for visits, better expression of ideas and concerns, and encouraging engagement in self-care and self-management [17,24,31,35].	Moderate	10xA	none	6xA
	Patient portals support communication, enhance discussions and shifts power relations between patients and providers [17,24,29,33,35].	Moderate	9xA	none	none
	Patient portals can improve quality of care and caregiver experience, and reduce care burden [17,31,33]	Low	4xA	none	1xA<br>1xD

[a] Indicates the confidence in the evidence, and was calculated using CERQual-UR (Grading of Confidence in the Evidence of Qualitative Research at the Level of an Umbrella Review) based on methodological limitations, coherence, relevance and adequacy. Ratings are from high, moderate, low, and very low.

[b] Quantitative descriptive findings originate from quantitative PSs for which SR authors: a) did not report statistical significance, or b) reported statistical significance, but did not have sufficient number of finding (i.e. greater than 2) to be included in GRADE-UR evaluation.
